# Reduction of central venous catheter associated blood stream infections following implementation of a resident oversight and credentialing policy

**DOI:** 10.1186/1754-9493-5-15

**Published:** 2011-06-03

**Authors:** Robert A Cherry, Cheri E West, Maria C Hamilton, Colleen M Rafferty, Christopher S Hollenbeak, Gregory M Caputo

**Affiliations:** 1 Department of Surgery, The Pennsylvania State University, College of Medicine, Hershey, PA, USA; 2 Clinical Quality Management & Performance Improvement, Penn State Milton S. Hershey Medical Center, Hershey, PA, USA; 3 Department of Medicine, The Pennsylvania State University, College of Medicine, Hershey, PA, USA; 4 Departments of Surgery and Public Health Science, The Pennsylvania State University, Hershey, PA, USA

## Abstract

**Background:**

This study assesses the impact that a resident oversight and credentialing policy for central venous catheter (CVC) placement had on institution-wide central line associated bloodstream infections (CLABSI). We therefore investigated the rate of CLABSI per 1,000 line days during the 12 months before and after implementation of the policy.

**Methods:**

This is a retrospective analysis of prospectively collected data at an academic medical center with four adult ICUs and a pediatric ICU. All patients undergoing non-tunneled CVC placement were included in the study. Data was collected on CLABSI, line days, and serious adverse events in the year prior to and following policy implementation on 9/01/08.

**Results:**

A total of 813 supervised central lines were self-reported by residents in four departments. Statistical analysis was performed using paired Wilcoxon signed rank tests. There were reductions in median CLABSI rate (3.52 vs. 2.26; p = 0.015), number of CLBSI per month (16.0 to 10.0; p = 0.012), and line days (4495 vs. 4193; p = 0.019). No serious adverse events reported to the Pennsylvania Patient Safety Authority.

**Conclusions:**

Implementation of a new CVC resident oversight and credentialing policy has been significantly associated with an institution-wide reduction in the rate of CLABSI per 1,000 central line days and total central line days. No serious adverse events were reported. Similar resident oversight policies may benefit other teaching institutions, and support concurrent organizational efforts to reduce hospital acquired infections.

## Introduction

Hospital acquired infections are a growing public health concern because of their impact on morbidity and mortality and their potential preventability. Central venous catheters (CVC) account for about 90 percent of catheter-related bloodstream infections (BSIs) [1]. As a result, there are somewhere between 500 and 4,000 patient deaths each year in the U.S. related to central line associated blood stream infections (CLABSI), with the cost per BSI estimated at $33,039 [2]. Reducing the rate of CLABSI became an organizational quality and patient safety goal in order to reduce morbidity, mortality, and health care-related costs.

The use of meticulous technique and evidence-based guidelines by experienced physicians has led to reductions in CLABSI at many facilities. One effective way to reduce these types of infections is to develop and implement a CLUE (Central Line Utilization Education) insertion bundle [3]. The CLUE insertion bundle consisted of several important steps related to central line insertion and maintenance. These include hand hygiene, maximal barrier precautions on insertion, chlorhexidine skin antisepsis, optimal catheter site selection, and daily review of line necessity.

However, there are inherent educational and patient safety challenges for academic medical centers that sponsor residency programs. Although our hospital has a CLUE insertion bundle, and monitors compliance with these guidelines, the effectiveness of the bundle has been questioned because of inconsistency in resident oversight during CVC insertions, and lack of integration with nursing practice guidelines. Bedside behavioral interventions have been shown to enhance the quality of compliance with best practice principles [4].

We therefore developed and implemented an institution-wide resident oversight and credentialing policy for CVC placement, and evaluated the impact that such a policy had on reducing the rate of CLABSI per 1,000 line days during the 12 months before and after implementation of the policy. We hypothesized that the development, implementation, and enforcement of a resident oversight and credentialing policy for CVC placement may result in a decrease in the rate of CLABSI, especially if it is fully integrated with the CLUE insertion bundle and accompanied by reinforcement of nursing practice guidelines.

## Materials and methods

### Study Setting

The Penn State Milton S. Hershey Medical Center consists of a 488-bed academic medical center, the College of Medicine, a biomedical research complex, an ambulatory surgery center, and numerous outpatient facilities. The PSMHC has joint ventures in rehabilitation medicine and psychiatry. There are a total of 48 residency and fellowship program directors in various medical and surgical specialties. There were 3.6 CLABSI per 1000 central line days in fiscal year 2008.

### Project Team

The project team consisted of selected residency program directors, an intensivist, and the leadership from the Department of Quality, including a clinical performance improvement specialist, the Associate Chief Quality Officer. The project team was charged by the Chief Quality Officer to develop a process and procedure for achieving resident oversight for all CVC insertions by an experienced attending, in collaboration with the educational strategies and practice guidelines developed and implemented by the hospital's CLUE Team.

### Study Design

This is a retrospective analysis of prospectively collected data at an academic medical center prior to and following policy implementation on 9/01/08. All adult and pediatric patients undergoing non-tunneled central venous line placement, and other percutaneously inserted central catheters, that are used for infusion and monitoring purposes from September 1, 2008 through August 31st, 2009 were included. This group was subject to the new CVC resident oversight and credentialing policy, and was compared to a control group that underwent CVC placement prior to policy implementation for the period September 1, 2007 thru August 31st 2008. Patients who had placement of a dialysis or apheresis catheter were excluded from the study.

Residents were required to have a simulated experience in CVC insertion, as determined by the clinical department. The CLUE insertion bundle slides and the New England Journal of Medicine (NEJM) training video was to be reviewed prior to the first insertion (no time constraint) and within 24 hours (before or after) of the second and third insertions. All residents were required to demonstrate competence by direct supervision for at least five (5) CVCs at a given anatomic site (internal jugular, subclavian, and femoral) prior to inserting lines independently at that site. The optimal site insertion in the CLUE bundle was considered the subclavian vein and femoral lines were to be avoided if possible. Ultrasound guided insertion was encouraged since it has been shown to reduce insertion-related complications and decrease the number of attempts required for successful insertion [5,6]. Insertion of central lines by any resident without supervision, even if deemed competent for independent insertion, were only allowed in urgent or emergent clinical situations.

Nurses also received hands-on training and a self-learning packet reinforcing existing central line insertion and maintenance practice by the CLUE project team. The self-learning packets also contained pre- and post-test questionnaires to strengthen the educational objectives, and support practice standards that were also being taught concurrently to residents. An internal education video designed to demonstrate the correct nursing practice in the care of central lines was also made available. Disparate policies on central venous catheter care were also integrated into a single document.

The following data were collected in the twelve months prior to and the twelve months following the start of the policy: 1) number of central venous catheter associated blood stream infections; 2) number of central line days; 3) central venous catheter associated bloodstream infections per 1000 central line days; 4) central line bundle compliance; and 5) number of nosocomial bloodstream infections, and 6) serious adverse events reportable under Pennsylvania's Act 13 (Medical Care Availability and Reduction of Error Act).

Residents were required to log their central line procedures into a database that allowed the supervising physician to electronically attest that all criteria for a successful central line insertion were met (New Innovations Inc, Uniontown, Ohio). The Physician and Nurse Champions in their respective units were accountable for measuring compliance with the insertion and maintenance bundles and reporting these results to the CLUE team. Historical control charts were used to visualize trends in the data collected. Differences between these data elements for the time periods identified were analyzed using two-tailed, Wilcoxon signed rank tests. Because we used nonparametric statistical tests, we report median rather than means. Statistically significant differences were determined using a p-value of less than 0.05. This study was approved by our Institutional Review Board.

### Project Milestones

**Figure 1:**
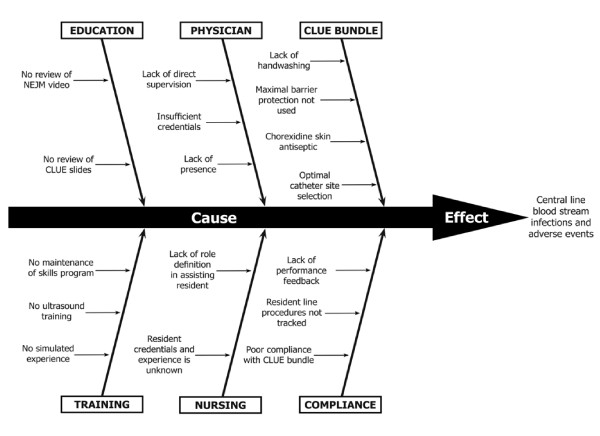
Cause and effect (Fishbone) diagram

A cause and effect (fishbone) diagram was developed by the Project Team to evaluate major deficiencies leading to CVC infections and to guide policy development (Figure 1). The timetable in which stakeholder approval was accomplished during this study is listed in Table 1. Institutional support, feedback, and buy-in was sought through presentation and approval of the various committees listed in the table. Each committee was requested to communicate the salient aspects of the policy to their respective constituency.

**Table 1:** Key milestones

Date	Stakeholder Policy Approval Timeline
1-Apr-08	Physician Champion/Project Leader Appointed
7-May-08	Clinical Chairs Council
14-May-08	Clinical Team
15-May-08	Risk Management
19-May-08	Patient Safety Committee
19-May-08	Quality Oversight Committee
3-Jun-08	Nursing Practice Council
16-Jun-08	Graduate Medical Education Committee
18-Jun-08	Medical Executive Committee Approval
21-Jul-08	Procedure/Credentialing Database completed
1-Sep-08	Policy Start Date/Data Collection
31-Dec-08	Nursing Education Completed

Cause and effect (Fishbone) diagram.

### Interventions

There were a number of interventions planned and implemented by the project team to enhance communication and achieve sustainability. The major interventions performed during the course of the study are as follows:

1) Review and reinforcement of the resident oversight policy through institution-wide e-mails and departmental meetings (August 2008 through March 2009).

2) Monitoring the documentation for credentialing through a database called New Innovations (October 2008 through September 2009).

3) "Tip of the Week" e-mail updates sent to all residents with instructions on how to correctly use the database (October 2008 thru March 2009).

## Results

### Data Analysis

During the 12 month period from September 2008 to August 2009, a total of 813 supervised central lines were self-reported by residents in four departments into the New Innovations Procedure Tracker database (Table 2). There were reductions in the median CLABSI rate (3.53 vs. 2.26; p = 0.015), the number of CLABSI per month (6.06 to 2.87; p = 0.012), and central line days (4495 vs. 4193; p = 0.019; **see **Tables 3, 4, and 5). Compliance measurements with the insertion bundle were discontinued by the CLUE team 4 months post intervention because substantial adherence to the protocol was achieved. We therefore do not believe that the modest and non-significant 1.1% increase in adherence was clinically relevant in reducing the CLABSI rate. (**see **Table 6).

**Table 2:** CVC insertions by department

Department	CVC insertions
Emergency Medicine	382
Medicine	230
Pediatrics	65
Surgery	136
Total	813

**Table 3:** Central line bloodstream infection rate

Central Line Bloodstream Infection Rate (CLABSI per 1000 central line days)			
**Months**	**FY 08**	**FY 09**	**Variance**

September	6.17	3.06	-101.63
October	5.37	3.92	-36.99
November	6.03	2.78	-116.91
December	4.78	1.70	-181.18
January	3.78	2.13	-77.46
February	3.32	3.08	-7.79
March	2.67	2.26	-18.14
April	2.15	1.15	-86.96
May	0.73	3.05	76.07
June	2.50	2.16	-15.74
July	3.72	1.16	-220.69
August	2.42	2.25	-7.56
Mean	3.64	2.39	
Median	3.52	2.26	
SD	1.68	0.83	
Lower IQR	2.46	1.92	
Upper IQR	5.08	3.06	

p-value = 0.015

SD = standard deviation; IQR = interquartile range

**Table 4:** Central line bloodstream infections

Central Line Bloodstream Infections (number of CLABSI)			
**Months**	**FY 08**	**FY 09**	

September	27.00	12.00	
October	28.00	15.00	
November	29.00	12.00	
December	22.00	7.00	
January	18.00	9.00	
February	14.00	13.00	
March	13.00	10.00	
April	9.00	5.00	
May	3.00	12.00	
June	11.00	9.00	
July	18.00	7.00	
August	10.00	10.00	
Mean	16.83	10.08	
Median	16.00	10.00	
SD	8.32	2.87	
Lower IQR	10.50	8.00	
Upper IQR	24.50	12.00	

p-value = 0.012

SD = standard deviation; IQR = interquartile range

**Table 5:** Central line days

Central Line Days (# central line days)			
**Months**	**FY 08**	**FY 09**	**Variance**

September	4376.00	3923.00	-11.55
October	5219.00	3830.00	-36.27
November	4808.00	4316.00	-11.40
December	4598.00	4114.00	-11.76
January	4758.00	4226.00	-12.59
February	4213.00	4224.00	0.26
March	4861.00	4443.00	-9.41
April	4190.00	4352.00	3.72
May	4090.00	3938.00	-3.86
June	4393.00	4146.00	-5.96
July	4842.00	4356.00	-11.16
August	4198.00	4448.00	5.62
Mean	4545.50	4193.00	
Median	4495.50	4225.00	
SD	353.56	207.74	
Lower IQR	4205.50	4025.00	
Upper IQR	4825.00	4354.00	

p-value = 0.019

SD = standard deviation; IQR = interquartile range

**Table 6:** Central line bundle compliance†

Central Line Bundle Compliance			
**Months**	**FY 08**	**FY 09**	**Variance**

September	96.0	100.0	4.0
October	97.0	100.0	3.0
November	980	100.0	2.0
December	98.0	100.0	2.0
January	98.0	97.0	-1.0
Mean	97.4	99.4	
Median	98.0	100.0	
SD	0.9	1.3	
Lower IQR	97.0	100.0	
Upper IQR	98.0	100.0	

†Monitoring for central line compliance was discontinued by the CLUE Team after January 2009 since benchmarks were consistently achieved

p-value = 0.078

SD = standard deviation; IQR = interquartile range

There were 570 CVC insertions in which the anatomic site was specified: internal jugular (245), femoral vein (202), and subclavian vein (123). Most subclavian vein insertions were performed in the emergency department (162/202; 80.2%). There were no serious adverse events reported to the Pennsylvania Patient Safety Authority.

## Discussion

### Interpretation and Context

We have demonstrated significant reductions in CLABSI following implementation of a CVC resident credentialing and oversight policy that was tightly integrated with nursing practice education and policy. However, we believe that physician and nurse practice integration was essential in achieving maximal benefit. The use of the NEJM training video and CLUE slides was an important adjunct in teaching proper technique and enhancing knowledge and compliance with the insertion bundle. Video-based training, for example, has been shown to increase compliance with sterile technique during CVC insertions [7]. This was especially important in achieving integration of nursing practice guidelines with resident performance.

The use of simulation-based training in resident instruction was considered an important strategy. Such training has been shown to decrease the number of needle passes during the performance of the procedure [8,9], and reduce the number of arterial punctures and CVC adjustments [9]. In addition, Barsuk and colleagues demonstrated a significant decrease in the number of CLABSI, from 3.2 infections per 1000 line days to 0.5 infections per 1000 line days, after the introduction of simulation-based training for residents rotating through an adult ICU [10]. In another study by Ramsey and colleagues, a resident training program was implemented that involved CVC insertion web-based instruction, simulation training, and an observed clinical skills exercise. Reductions of central line associated BSIs were also observed [11]. The cost for annual simulation training can be expensive. Cohen and associates estimated that the annual cost for simulation training in CVC insertions was about $112,000 at their institution [12]. However, there was a net savings of approximately $700,000 in the MICU due to reductions in CLABSI.

Pronovost et. al. studied 108 ICU's in 67 hospitals that adopted similar evidence-based guidelines for reducing the CLABSI rate. This group of hospitals eliminated central line infections 3 months after the intervention and was sustained at 18 months. The authors acknowledged the potential for under-reporting in their study, which is not an issue in our single center investigation. Furthermore, the questions of how to implement sustainable evidence-based strategies in the teaching environment starts to emerge. Teaching hospitals composed 52% of the facilities in the Pronovost study. Our study may serve as a useful roadmap for the implement of best practices among residents working in teaching hospitals [13].

Of note, compliance measurements with the insertion bundle were discontinued by the CLUE team 4 months post intervention because substantial adherence to the protocol was achieved. We therefore do not believe that the modest 1.1% increase in adherence was clinically relevant in reducing the CLABSI rate.

Finally, common technical errors can probably be reduced if experienced mentors are available for teaching [14]. In one study involving the SICU, there was also a significant decrease in CLABSI following close supervision by an intensivist [15]. Faculty presence is therefore necessary to insure optimal technique, reinforce best practice principles, and demonstrate partnership with bedside nursing. Mentorship is also required to achieve the kind of behavioral changes needed to effect change at the bedside.

### Study Limitations

There are several limitations related to this study. First, the baseline data for resident-driven CVC insertions in the year prior to policy implementation is unknown. Professional charges may not capture the number of central lines inserted by residents unless there was an attending present for the critical portion of the procedure. Central supply records of central line kits may not be accurate if multiple CVC line kits were used on selected patients, or if the central line placement was unsuccessful. Infection control internally reports the rate of CLABSI based on the number of infections per 1000 line days. This is consistent with CDC criteria. However, we do not know the infection rate per CVC line inserted using this methodology.

Second, there is the possibility of a Hawthorne effect in which the desired behavior is improved based on the fact that the actions of providers involved in the procedure are being evaluated throughout the organization. This is an inherent limitation with many performance improvement projects because the interventions are typically not blinded and may be open to bias. Continued monitoring and feedback is required to insure sustainability of our intervention and hardwiring into the organizational culture.

Third, as with many institutions, concurrent interventions designed to achieve performance improvement may make interpretation of the data challenging, especially with respect to the relative impact that each one has on the primary metric. For instance, the project team intentionally capitalized on the enhancements to nursing practice in order to provide an integrated, team approach to reducing CLABSI. During this time, Nursing took disparate central line care policies throughout the institution and integrated them into a single policy. However, this policy and the associated training for all clinical service lines were not completed until December of 2008.

Fourth, there was a blood culture policy developed that provided for a standardized practice in obtaining blood cultures when using a CVC as the access source. However, this policy was not implemented until 10 months after the CVC resident oversight policy in April 2009, long after significant trends were realized in the CLABSI rate, and was not related to the reduction in central lines days.

Fifth, coordination of concurrent interventions along a predetermined launch date is not always possible. In addition to the blood culture policy, there was an internal education video designed to demonstrate the correct nursing practice in the care of central lines. This video was made available to nursing in June of 2008, approximately three months before implementation of the CVC resident oversight. Nurses also received hands on training and a self learning packet on central line care that was not completed until December 2008. Furthermore, clinical departments were already involved in simulated training of residents in July and August of 2008, along with reviews of the NEJM video and CLUE slides, in order to be compliant with the new policy starting in September of 2008. No other concurrent interventions overlapped during the 12-month period from September 2008 to August 2000.

Sixth, there was a change in definition for CLABSI in January of 2008. This may explain the decrease in the CLABSI rate from December 2007 to January 2008. However, the continued decline in the CLABSI rate that occurred throughout the rest of the year seems unlikely to be the result of a simple definition change.

Seventh, the CLABSI rate includes pediatric patients in which central line insertions tend to be performed by the supervising attending. However, the pediatric population accounted for only 8.0% of the reported central line insertions (65/813). The improvement in the overall CLABSI rate is therefore predominantly from the adult population.

Finally, there is no data available through pre- and post-test questionnaires to assess whether there was a short-term increase in knowledge gained as a result of these educational initiatives. Nevertheless, it would be difficult to demonstrate in this study whether or not short term gains in knowledge and skills, translates into long term outcome improvement. The increased faculty presence during the study period would be a complicating variable.

Nevertheless, we believe that these educational efforts were contributory to the reduction in central line rates. Prevention education programs that involve updates to written policies, self-study modules with pre- and post-tests, an didactic lectures have already been shown to reduce CLABSI [16]. We also know that a multidisciplinary education program directed at both nurses and physicians, and highlighting the correct practices for the reduction of CLABSI, can significantly decrease the infection rate [17]. In fact, Pronovost and associates have shown that multifaceted interventions may be required to achieve reductions in CLABSI [18]. Among the strongest interventions known to reduce CLABSI [1], full barrier precautions had already been implemented, and the practice of routine replacement of CVCs was already in place prior to the study period.

With that being said, the CVC resident oversight policy integrated concurrent efforts in a team-oriented approach by 1) asking the nurse to be present for bedside central line insertions and to help oversee and monitor the quality of compliance with the insertion bundle; 2) providing nursing access to the database regarding the central line experience for each resident; and 3) creating an environment that allowed the nurse to question the skill level of the physician performing the procedure. Nursing education, engagement, and willingness to change central line practice was therefore essential.

### Barriers to Improvement

There were several potential barriers to improvement that were recognized early during the project's development and implementation:

1) Resident and faculty compliance with the policy.

2) Faculty commitment to provide oversight and education during CVC insertions.

3) Faculty availability for resident education and supervision during nighttime hours

4) Faculty and resident documentation of the procedure for the purposes of house staff credentialing.

These changes in organizational culture were partly overcome through a coordinated communication plan (see *Interventions *above), engagement of the clinical chairs and program directors, and a commitment by the senior leadership of the hospital. Nevertheless, despite the impressive number of supervised central line insertions at our institution, residents and faculty in multiple departments have stated that the multistep process required for credentialing has been cumbersome. In addition, the number of faculty trained and competent in the use of ultrasound-guided CVC insertion has been a challenge. Finally, delegating the content of the simulation-based training program to the individual Department poses a potential barrier to standardized teaching methods and use of equipment. The American College of Surgeons and the Association of Program Directors in Surgery, for example, have recognized that lack of standardization in simulation training is a major obstacle to its teaching effectiveness [19].

### Lessons Learned

There were several lessons learned during the course of the performance improvement project that are worth mentioning should other institutions consider a similar strategy:

1) Ongoing feedback from vested stakeholders was necessary to achieve buy-in and perform effective policy revisions.

2) Early monitoring of New Innovations database is necessary to understand barriers to documentation and initiate steps to correct problems.

We also concluded that emergency department education was needed to decrease the number of femoral vein insertions and potential risk for infection. In addition, non-surgeons tended to use the internal jugular vein which contributed to the relatively low use of the subclavian site (21.6%). Site selection appeared to be influenced by prior experience and education. Future training efforts would therefore need to teach and enhance this important skill.

## Conclusions

Implementation of a new CVC resident oversight and credentialing policy has been significantly associated with an institution-wide reduction in the rate of CLABSI per 1,000 central line days and total central line days. No serious adverse events were reported. Similar resident oversight policies may benefit other teaching institutions, especially if it is supportive and in alignment with concurrent nursing efforts to reduce hospital acquired infections.

## Competing interests

The authors declare that they have no competing interests.

## Authors' contributions

All authors have read and approved the final manuscript. RAC carried out the conception, design, and the acquisition, analysis and interpretation of data, as well as the drafting of the manuscript. CEW and MCH participated in the acquisition, analysis and interpretation of data, and critically revised the manuscript for important content. CMR was involved in the interpretation of data and critical revisions of the manuscript for important content. CSH participated in the analysis and interpretation of data, and critically revised the manuscript for important content. GMC was involved in the interpretation of data and critical revisions of the manuscript for important content.
